# CSF and Brain Indices of Insulin Resistance, Oxidative Stress and Neuro-Inflammation in Early versus Late Alzheimer’s Disease

**DOI:** 10.4172/2161-0460.1000128

**Published:** 2013-10-31

**Authors:** Sarah Lee, Ming Tong, Steven Hang, Chetram Deochand, Suzanne de la Monte

**Affiliations:** 1 Department of Medicine, Rhode Island Hospital, Warren Alpert Medical School, Providence, RI, USA; 2 Department of Medicine, Warren Alpert Medical School, Providence, RI, USA; 3 Departments of Medicine, Rhode Island Hospital, Brown University, Providence, RI, USA; 4 Department of Medicine, Pathology (Neuropathology), Neurology and Neurosurgery, Rhode Island Hospital, Warren Alpert Medical School, Brown University, Providence, RI, USA

**Keywords:** Biomarkers, Cerebrospinal fluid, Neurotrophins, Amyloid, Tau, Insulin resistance, Neurodegeneration, Glucose metabolism, Luminex

## Abstract

Alzheimer’s disease (AD) is characterized by progressive impairments in cognitive and behavioral functions with deficits in learning, memory and executive reasoning. Growing evidence points toward brain insulin and insulin-like growth factor (IGF) resistance-mediated metabolic derangements as critical etiologic factors in AD. This suggests that indices of insulin/IGF resistance and their consequences, i.e. oxidative stress, neuro-inflammation, and reduced neuronal plasticity, should be included in biomarker panels for AD. Herein, we examine a range of metabolic, inflammatory, stress, and neuronal plasticity related proteins in early AD, late AD, and aged control postmortem brain, postmortem ventricular fluid (VF), and clinical cerebrospinal fluid (CSF) samples. In AD brain, VF, and CSF samples the trends with respect to alterations in metabolic, neurotrophin, and stress indices were similar, but for pro-inflammatory cytokines, the patterns were discordant. With the greater severities of dementia and neurodegeneration, the differences from control were more pronounced for late AD (VF and brain) than early or moderate AD (brain, VF and CSF). The findings suggest that the inclusion of metabolic, neurotrophin, stress biomarkers in AβPP-Aβ+pTau CSF-based panels could provide more information about the status and progression of neurodegeneration, as well as aid in predicting progression from early- to late-stage AD. Furthermore, standardized multi-targeted molecular assays of neurodegeneration could help streamline postmortem diagnoses, including assessments of AD severity and pathology.

## Introduction

Growing evidence supports the concept that in Alzheimer’s disease (AD), metabolic dysfunction, mediated by impairments in insulin and insulin-like growth factor (IGF) signaling [1–12], causes progressive deficits in brain glucose utilization, energy metabolism, cytoskeleton and myelin maintenance, and neuronal plasticity [13,14]. Further contributions from oxidative and endoplasmic reticulum stress, inflammation, and increased pro-death/anti-survival signaling, help drive neurodegeneration. Consequences include brain accumulations of amyloid beta (AβPP-Aβ) deposits and fibrils/oligomers, and phospho-tau-related neuronal cytoskeletal lesions [13–16]. In addition, insulin resistance down-regulates target genes needed for cholinergic function, further compromising neuronal plasticity, memory, and cognition [13,14]. The pivotal roles of insulin and IGF-1 resistance as mediators of cognitive impairment and neurodegeneration have been well documented in humans and experimental animals [7,11,12,17]. This concept is corroborated by the findings that cognitive impairment and neurodegeneration can be slowed, reduced in severity, or prevented in experimental animals and humans by treatment with insulin sensitizer agents, insulin, or long-acting glucagon-like peptide-1 (GLP-1)-related compounds [18–27].

Over the past two decades, robust efforts to develop non-invasive diagnostic assays for AD have led to protocols that measure cerebrospinal fluid (CSF) levels of Tau, hyperphosphorylated Tau (pTau) [28–31] and AβPP-Aβ [32–38], and positron emission tomography (PET) to image aberrant brain accumulations of AβPP-Aβ [39]. When combined with magnetic resonance imaging (MRI), functional MRI (fMRI), and PET studies of brain glucose utilization, CSF assays of Tau, pTau and AβPP-Aβ correlate well with intermediate and late stages of AD [33,40]. However, without the support of costly neuroimaging studies, the sensitivity, specificity, and reproducibility of highly restricted CSF-based assays are not sufficient to serve as stand-alone diagnostic aids or measures of treatment responses. On the other hand, PET studies of brain glucose metabolism and other means of assessing brain metabolic dysfunction, should be incorporated into AD diagnostic and assessment plans since the related abnormalities occur early and progress with severity of disease. Besides the proteins that directly pertain to insulin and IGF-1 resistance, i.e. the trophic factors/ligands themselves, associated molecules that further contribute to neurodegeneration, i.e. indices of oxidative stress, inflammation, and impaired neuronal plasticity, should be evaluated [41–43]. Here in, we examine the potential value of using CSF-based multi-pronged platforms for AD diagnosis and monitoring. The goal was to determine the degree to which indices of insulin/IGF resistance, neuroinflammation, oxidative stress, and neuronal plasticity in CSF correspond with those in postmortem ventricular fluid (VF) and brain tissue.

## Methods

### Human brain tissue, VF, and CSF

Fresh frozen samples of postmortem frontal lobe (Brodmann Area 8/9) from patients with no evidence of AD (Braak Stage 0–1), moderate AD (Braak States 3–4), or advanced AD (Braak Stage 6), were obtained from the Kathleen Price Bryan Brain Bank at Duke University Medical Center, the Massachusetts General Hospital Alzheimer’s Disease Research Center Brain Bank, and the Brown University Brain Bank [11,12,16,44]. With regard to #1 and #2, the subject in all groups had similar mean ages and specra of underlying diseases that contributed to death as described earlier [11,12,16,44]. The gender ratios were approximately even in control and moderate AD, but skewed with 75% female in the advanced AD (Braak 6) group as reported earlier [44]. Postmortem intervals were less than 14 hours and the tissues were stored at −80°C. Aqueous homogenates of brain tissue were used to obtain cytosolic and extracellular fluid proteins for comparison with VF and CSF results. To obtain these, brain tissue samples were homogenized in 5-volumes of phosphate-buffered saline (PBS; 10 mM phosphate, 0.9% sodium chloride, pH 7.35) containing 0.02% sodium azide and protease inhibitors [11,12]. Supernatants obtained by centrifuging the samples at 12000 × g for 10 minutes at 4°C were used in the studies. Protein concentrations were measured with the Nano-Orange Protein Quantification Kit (Pierce Chemical Company, Rockford, IL).

Postmortem VF was obtained from the Massachusetts General Hospital’s Alzheimer’s Disease Research Center Brain Bank. CSF samples were obtained for diagnostic or research purposes from patients fulfilling NINDS criteria for probable or definite AD (N=16) [45]. Control CSF samples were from patients undergoing evaluation for headache or back pain, and who were free of neoplastic, inflammatory and neurodegenerative diseases (N-16). CSF was obtained by lumbar puncture and stored at −80°C. Research CSF samples were obtained by written informed consent using guidelines set by the Human Studies Committees at the National Institutes of Health. All samples were de-identified and their use in this study was approved by the Lifespan Human Studies Committee and Investigational Review Board.]

### Enzyme-linked immunosorbent assay (ELISA)

Aqueous anterior frontal lobe homogenates, VF, and CSF samples were serially diluted in PBS containing 1% bovine serum albumin (PBS-BSA). Immunoreactivity to insulin, nerve growth factor (NGF), brain derived neurotrophic factor (BDNF), glial cell derived neurotrophic factor (GDNF), phospho-tau, AβPP, AβPP-Aβ, 8-hydroxydeoxyguanosine (8-OHdG), 4-hydroxynonenal (4-HNE), and advanced glycation end-products (AGE) was measured by direct binding ELISA using with horseradish peroxidase (HRP)-conjugated secondary antibody and Amplex Ultra Red soluble fluorophore [16,46]. Fluorescence intensity was measured (Ex 565 nm/Em 595 nm) in a Spectra Max M5 microplate reader (Molecular Devices, Sunnyvale, CA). All assays were performed in quadruplicate. Binding specificity was determined by omitting primary or secondary antibodies in parallel reactions. Immunoreactivity was normalized to protein content.

### Multiplex ELISA

We used Luminex bead-based multiplex ELISA’s to measure immunoreactivity to pro-inflammatory cytokines, chemokines, growth factors, and insulin-related gut hormones (Millipore or Bio-Rad; Table 1). After re-centrifuging the brain, VF, and CSF samples to remove particulate debris (12000×g/10 minutes, 4°C), the supernatants were filtered (0.45 μm pores). For these assays, the samples were diluted in PBS (brain) or used undiluted (VF and CSF). Samples (200 μl) were incubated with the beads, and captured antigens were detected with biotinylated secondary antibody and phycoerythrin-conjugated Streptavidin according to the manufacturer’s protocol. Immunoreactivity was measured in a Bio-Plex 200 system (Bio-Rad, Hercules, CA). Data are expressed as fluorescence light units corrected for protein concentration.

### Statistical analysis

Inter-group comparisons of brain tissue results were made by analysis of variance (ANOVA) with linear trend and Fisher LSD post-hoc tests. VF and CSF AD versus control comparisons were made using Student *t*-tests. Box plots reflect group medians (horizontal bar), 95% confidence interval limits (upper and lower box limits) and range (whiskers). Data were analyzed using GraphPad Prism 6 software (GraphPad Software, Inc., San Diego, CA).

## Results

### AD and oxidative stress markers in postmortem brains

**Figure 1:**
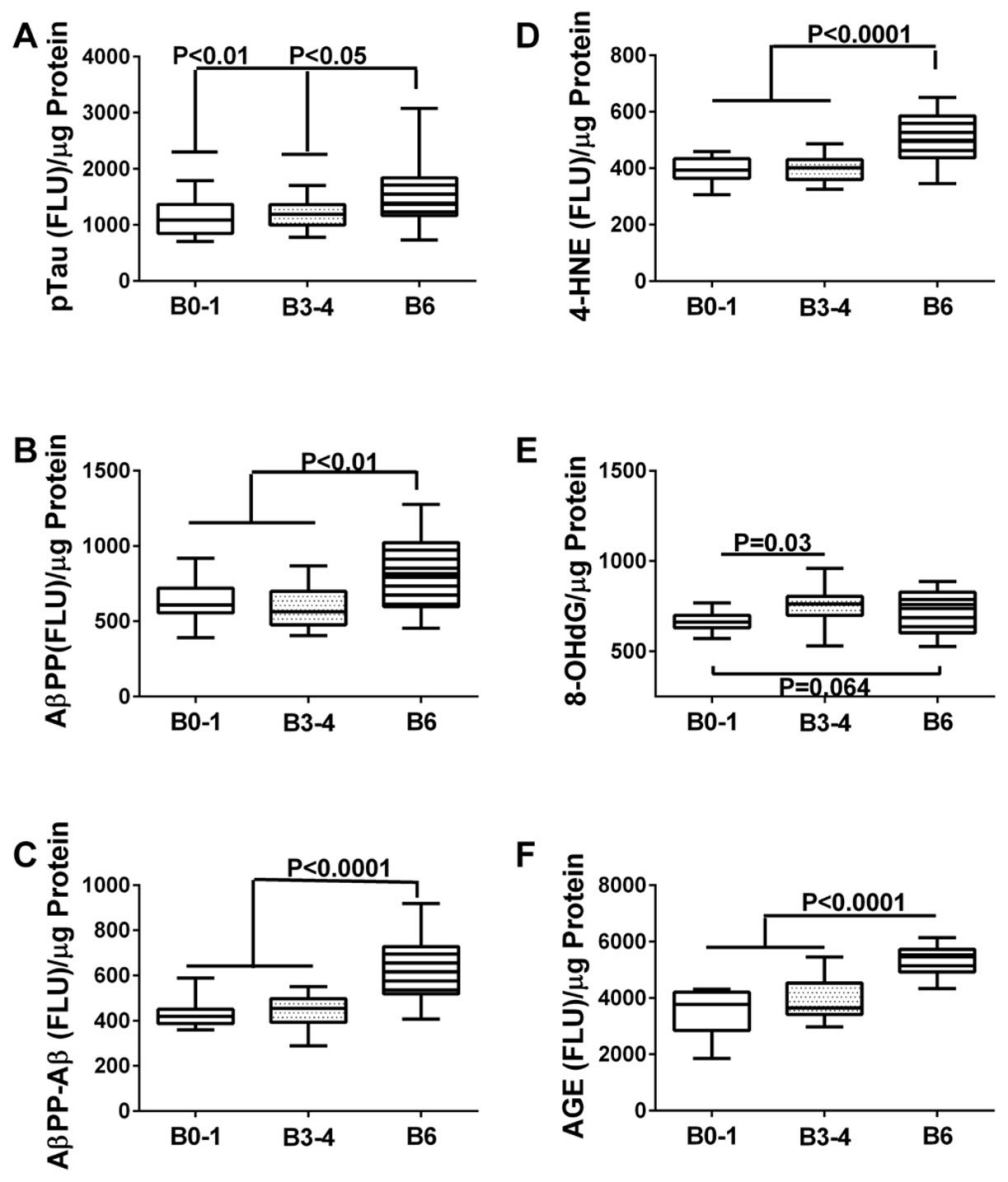
Biomarker indices of AD and oxidative stress in postmortem brains Frontal lobe aqueous homogenates from subjects with Braak Stage (B) 0–1 (controls), B3–4 (moderate AD), or B6 (late AD) pathology (N=8/group) were used to measure (A) pTau, (B) amyloid precursor protein (AβPP), (C) amyloid precursor protein-Abeta (AβPP-Aβ), (D) 4-hydroxynonenol (HNE), (E) 8-hydroxydeoxyguanosine (8-OHdG), and (F) advanced glycation end-product (AGE) by direct binding ELISA. Immunoreactivity was detected with HRP-conjugated secondary antibody and Amplex Red soluble fluorophor. Fluorescence light units (FLU) were measured (Ex 579 nm/Em 595 nm) in a Spectromax M5, and results were normalized to sample protein content. Results were analyzed by one-way repeated measures ANOVA with post hoc Fisher tests.

Direct-binding ELISA results for pTau, AβPP, AβPP-Aβ, 4-HNE, 8-OHdG, and AGE were analyzed with respect to Braak AD stage (Figure 1). One-way ANOVA tests demonstrated significant intergroup differences with respect to pTau (F=5.115; P=0.01), AβPP (F=7.47; P=0.0016), AβPP-Aβ (F=19.66; P<0.0001), 4-HNE (F=14.88; P<0.0001), and AGE (F=28.39; P<0.0001), and a significant trend for 8-OHdG (F=2.91; P=0.065). Post-hoc Fisher tests demonstrated higher levels of pTau (Figure 1A), AβPP (Figure 1B), AβPP-Aβ (Figure 1C), 4-HNE (Figure 1D), and AGE (Figure 1F) in AD Braak Stage 6 relative to the other groups. In contrast, 8-OHdG immunoreactivity was significantly elevated in Braak 3–4 AD brains, and only modestly increased at Braak 6 (Figure 1E) relative to control. This suggests that elevated levels of 8-OHdG may mark early to intermediate stages of neurodegeneration, and that a more sensitive assay may be required to assess DNA damage in late stages of disease. In addition, we observed a significant linear trend for increasing AGE levels and AD severity (F=28.39; P<0.0001), consistent with previous observations [47,48]. AGE marks oxidation related to insulin resistance.

### Insulin resistance and related proteins

**Figure 2:**
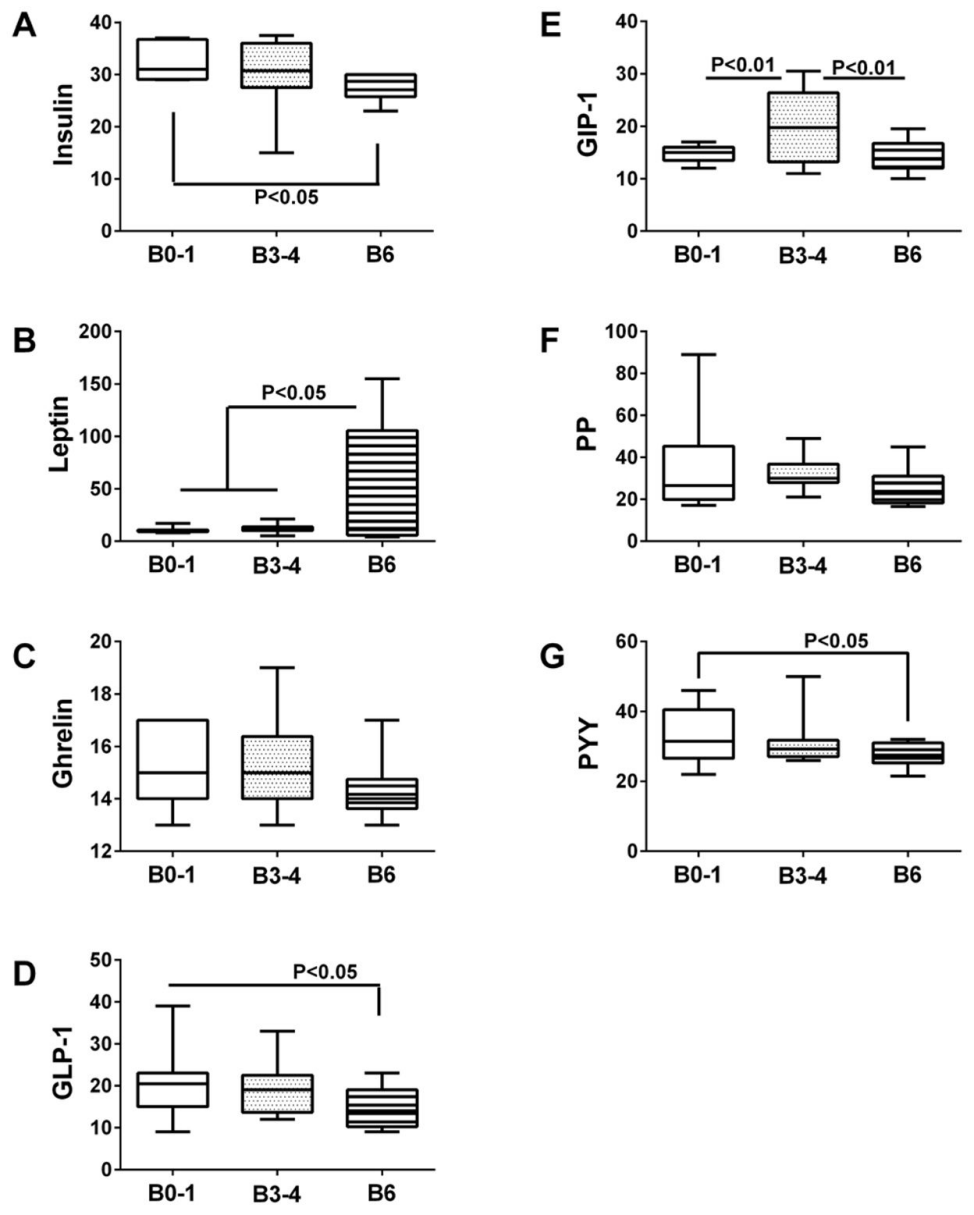
Insulin resistance biomarkers in brain Frontal lobe aqueous homogenates from subjects with Braak Stage (B) 0–1 (controls), B3–4 (moderate AD), or B6 (late AD) pathology (N=8/group) were used to measure (A) insulin, (B) leptin, (C) ghrelin (D) GLP-1, (E) GIP-1, (F) PP, and (G) PYY by multiplex bead-based ELISA. Immunoreactivity is expressed in fluorescence light units (FLU) normalized to protein content. Data were analyzed by one-way repeated measures ANOVA with post hoc Fisher significance tests.

**Figure 3:**
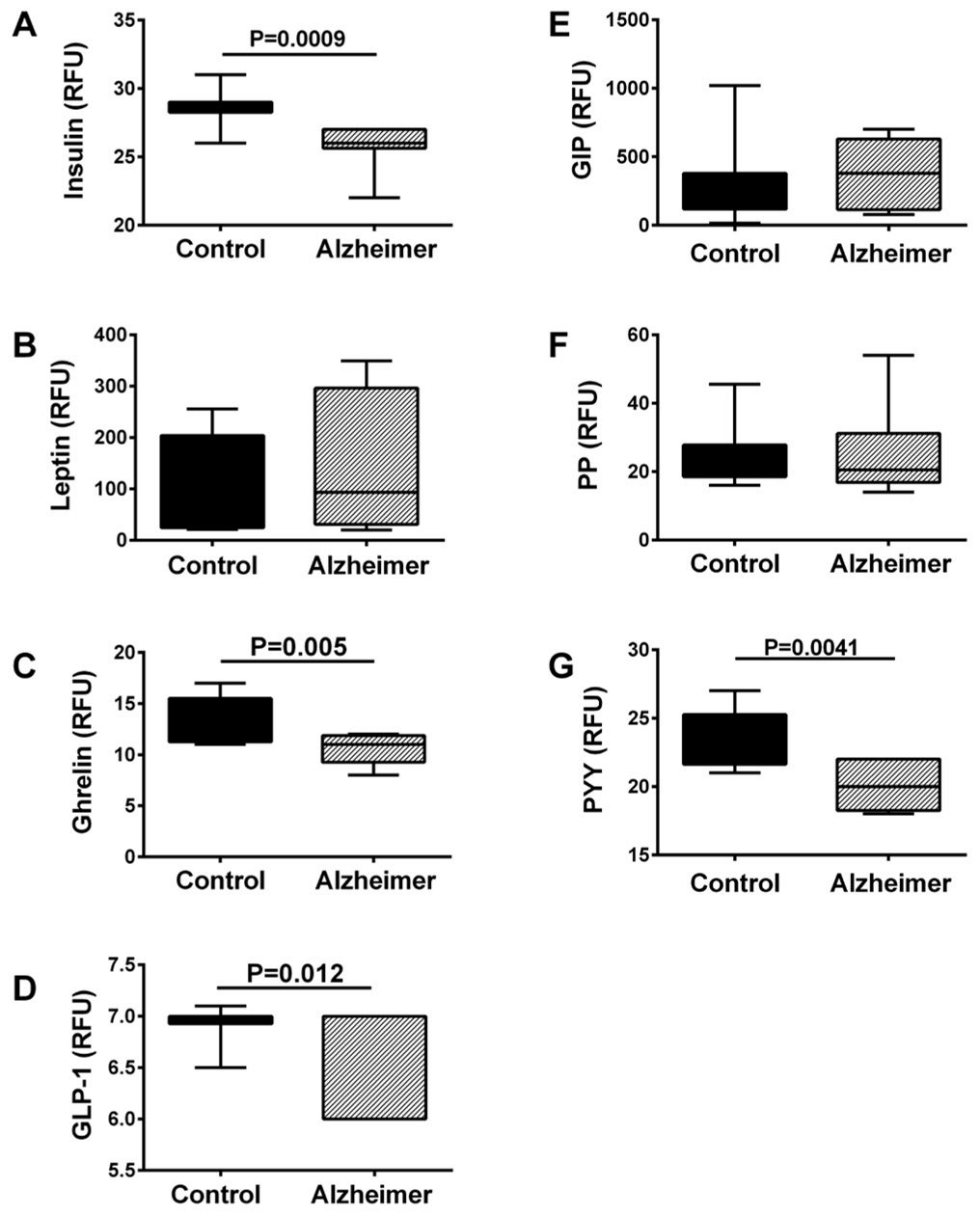
CSF indices of insulin resistance Lumbar puncture CSF from controls (N=12) and patients with early probable AD (N=16) were used to measure (A) insulin, (B) leptin, (C) ghrelin (D) GLP-1, (E) GIP-1, (F) PP, and (G) PYY by multiplex bead-based ELISA. Immunoreactivity is expressed in fluorescence light units (FLU) normalized to protein content. Data were analyzed with Student T tests.

A Gut Hormone Multiplex ELISA panel was used to measure insulin, leptin, ghrelin, glucagon-like peptide-1 (GLP-1), gastric inhibitory polypeptide (GIP-1), pancreatic polypeptide (PP), and Peptide tyrosine tyrosine (PYY) in brain tissue (Figure 2) and CSF (Figure 3). For postmortem brain tissue, ANOVA tests demonstrated significant inter-group differences with respect to insulin (F=3.305; P=0.046), GLP-1(F=3.47; P=0.041), GIP-1 (F=7.45; P=0.0016), leptin (F=5.38; P=0.008), and PYY (F=3.04; P<0.05). Post-hoc Fisher tests revealed significantly reduced levels of insulin (Figure 2A), GLP-1 (Figure 2D), and PYY (Figure 2G), and increased levels of leptin (Figure 2B) at Braak Stage 6 relative to control. In addition, GIP-1 immunoreactivity was significantly elevated at Braak Stage 3–4 relative to control and Braak 6 brains (Figure 2E). Furthermore, trend line post-hoc tests demonstrated significant progressive declines in brain insulin (P=0.017), GLP-1 (P=0.016), and PYY (P=0.02) with increasing AD stage, AD stage-associated trend reductions in ghrelin (P=0.089) and PP (P=0.087), and significant increases in leptin (P=0.005) with AD severity. Analysis of CSF samples revealed significantly lower levels of insulin (Figure 3A), ghrelin (Figure 3C), and GLP-1 (Figure 3D) in subjects with probable AD relative to normal aging, but no significant inter-group differences with respect to leptin (Figure 3B), GIP-1 (Figure 3E), PP (Figure 3F), or PYY (Figure 3G). Therefore, concordant results between postmortem brain and clinical CSF assays were obtained with respect to insulin, GLP-1, and to some extent, ghrelin.

### Trophic factors

**Figure 4:**
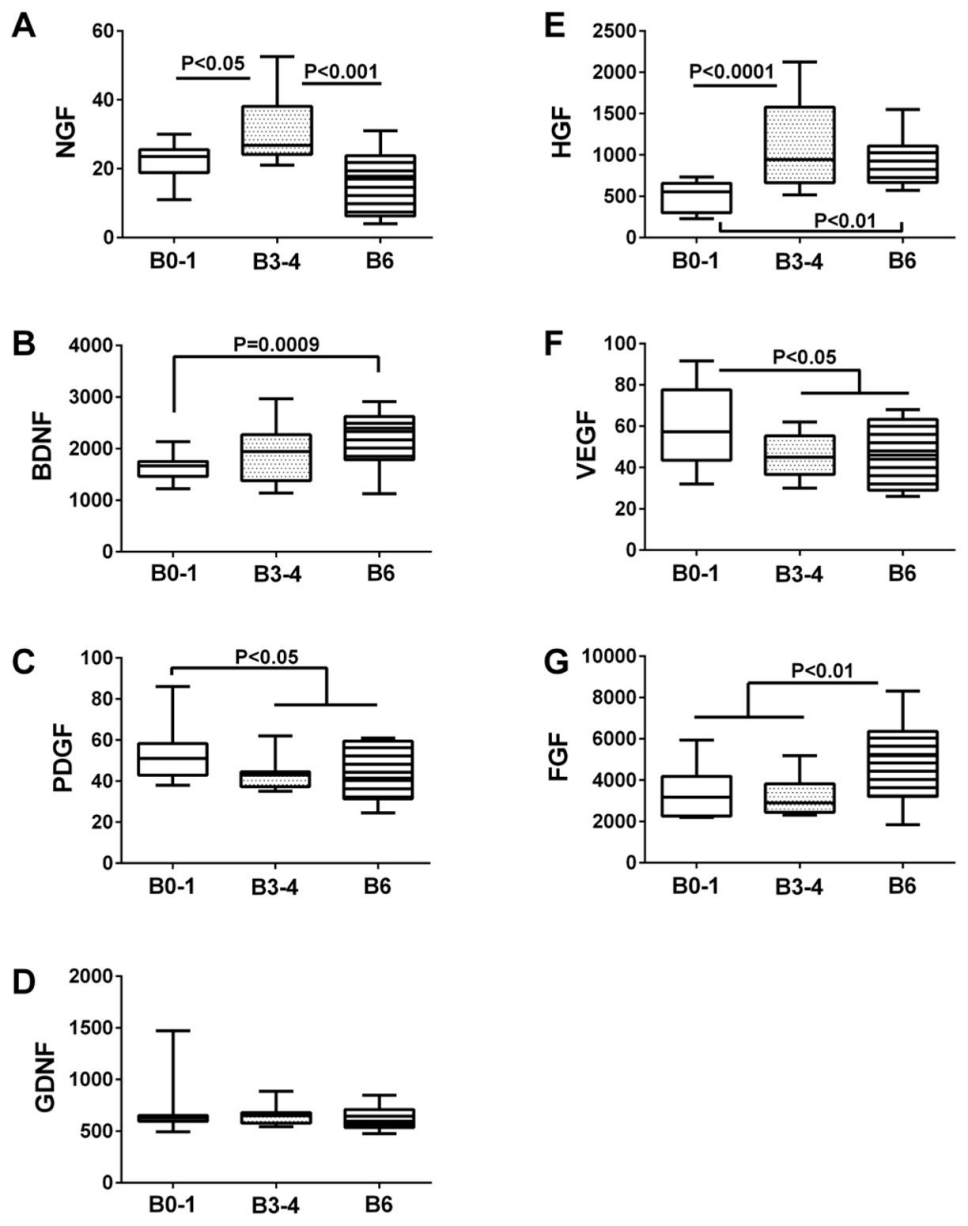
Trophic factor measurements in postmortem brains Frontal lobe aqueous homogenates from subjects with Braak Stage (B) 0–1 (controls), B3–4 (moderate AD), or B6 (late AD) pathology (N=8/group) were used to measure (A) NGF, (B) BDNF, (C) PDGF-AA, (D) glial cell derived neurotrophic factor (GDNF), (E) hepatocyte growth factor (HGF), and (F) basic fibroblast growth factor (FGF) by direct binding ELISA. Immunoreactivity was detected with HRP-conjugated secondary antibody and Amplex Red soluble fluorophor. Fluorescence was measured (Ex 579 nm/Em 595 nm) in a Spectromax M5, and results were normalized to sample protein content. Data were analyzed by one-way repeated measures ANOVA with post hoc Fisher significance tests.

Immunoreactivity to nerve growth factor (NGF), platelet-derived growth factor (PDGF), hepatocyte growth factor (HGF), vascular endothelial growth factor (VEGF), and fibroblast growth factor (β-FGF) were measured in brain (Figure 4), VF (Table 2), and CSF (Table 3) by multiplex ELISA. In addition, brain derived neurotrophic factor (BDNF) and glial derived neurotrophic factor (GDNF) were measured in brain homogenates by direct binding ELISAs (Figure 4). ANOVA tests demonstrated significant inter-group differences in postmortem brain levels of NGF (F=12.85; P<0.0001), BDNF (F=6.26; P=0.004), PDGF (F=3.624; P=0.035), HGF (F=10.08; P=0.0002), VEGF (F=3.54; P=0.037), and β-FGF (F=6.95; P=0.0023) (Table 2). Post-hoc trend line analysis demonstrated significant AD Braak stage dependent increases in BDNF (P=0.0009), HGF (P=0.0044), and β-FGF (P=0.0028), and decreases in PDGF (P=0.026) and VEGF (P=0.031). Fisher post-hoc inter-group comparisons demonstrated significantly higher NGF levels in Braak 3–4 compared with control and Braak 6 brains (Figure 4A), higher BDNF levels in Braak 6 relative to control (Figure 4B), HGF levels in Braak 3–4 and Braak 6 relative to control (Figure 4E), and β-FGF in Braak 6 relative to both control and Braak 3–4 brains (Figure 4G). In contrast, brain PDGF (Figure 4C) and VEGF (Figure 4F) levels were significantly lower at Braak 3–4 and Braak 6 relative to control. GDNF protein levels did not change in relation to AD Braak stage (Figure 4D).

In postmortem VF samples, we detected significantly lower levels of PDGF (P=0.0002), VEGF (P=0.0003), and β-FGF (P=0.0005), and higher levels of HGF (P=0.0001) in AD (Braak Stage 5–6) relative to control (Table 2). In CSF from patients diagnosed with probable AD (confirmed by postmortem examination), VEGF (P<0.0001) levels were increased while β-FGF (P=0.025) levels were reduced relative to normal aged controls (Table 3). In addition, a trend for reduced NGF (P=0.10) in AD CSF was observed. The slightly higher CSF levels of HGF in the AD group were not statistically significant. Note that the VF and CSF results were concordant with respect to HGF and β-FGF, but discordant with respect to β-NGF, PDGF, and VEGF, indicating that the trends in trophic factor expression/secretion may shift with AD progression.

### Neuro-inflammation

**Figure 5:**
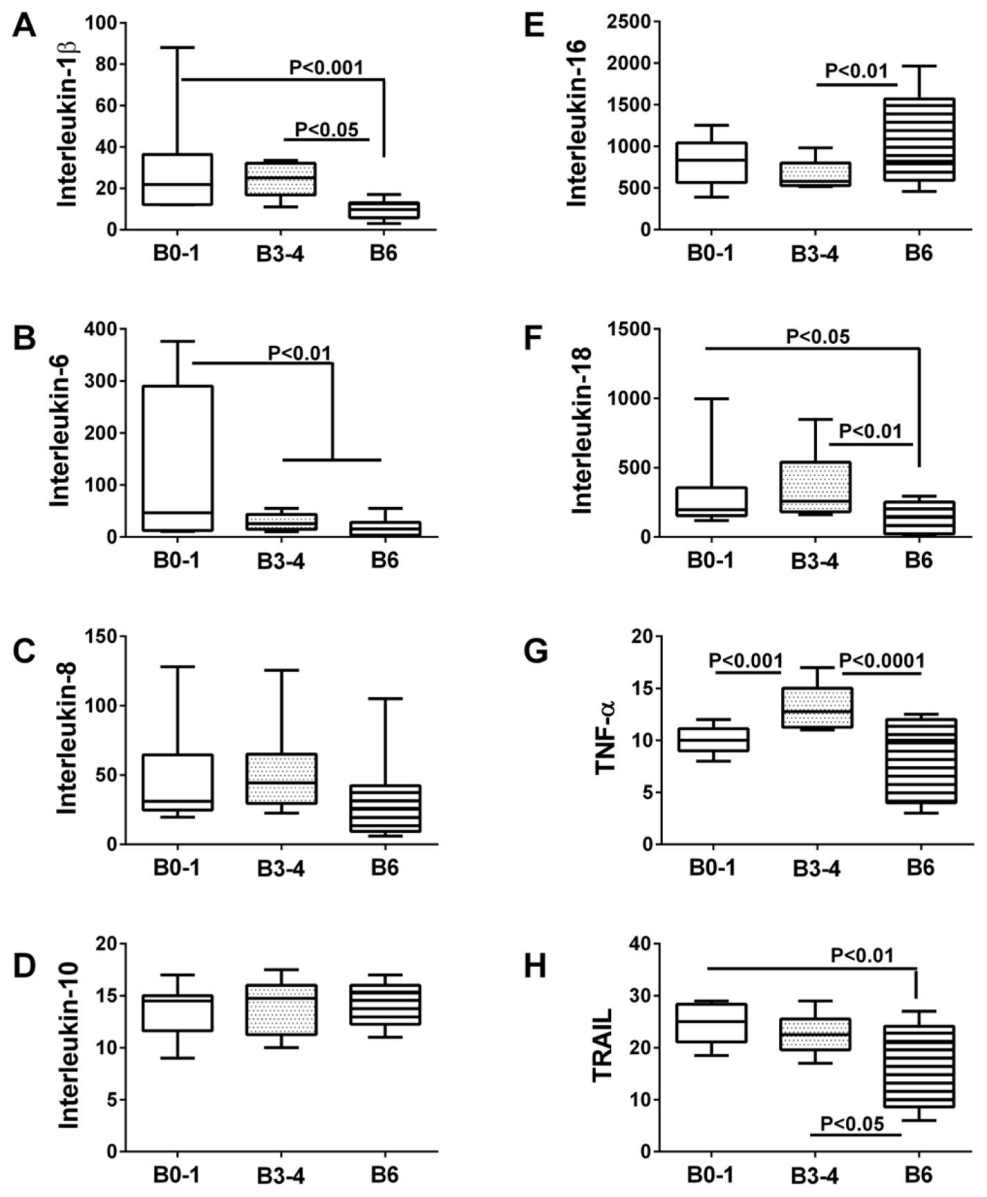
Inflammatory mediators in postmortem brains-1 Frontal lobe aqueous homogenates from subjects with Braak Stage (B) 0–1 (controls), B3–4 (moderate AD), or B6 (late AD) pathology (N=8/group) were used to measure (A) Interleukin-1β, (B) Interleukin-6, (C) Interleukin-8, (D) Interleukin-10, (E) Interleukin-16, (F) Interleukin-18, (G) Tumor necrosis factor-α (TNF-α), and (H) TRAIL by multiplex bead-based ELISA. Immunoreactivity is expressed in fluorescence light units (FLU) normalized to protein content. Data were analyzed by one-way repeated measures ANOVA with post hoc Fisher significance tests.

**Figure 6:**
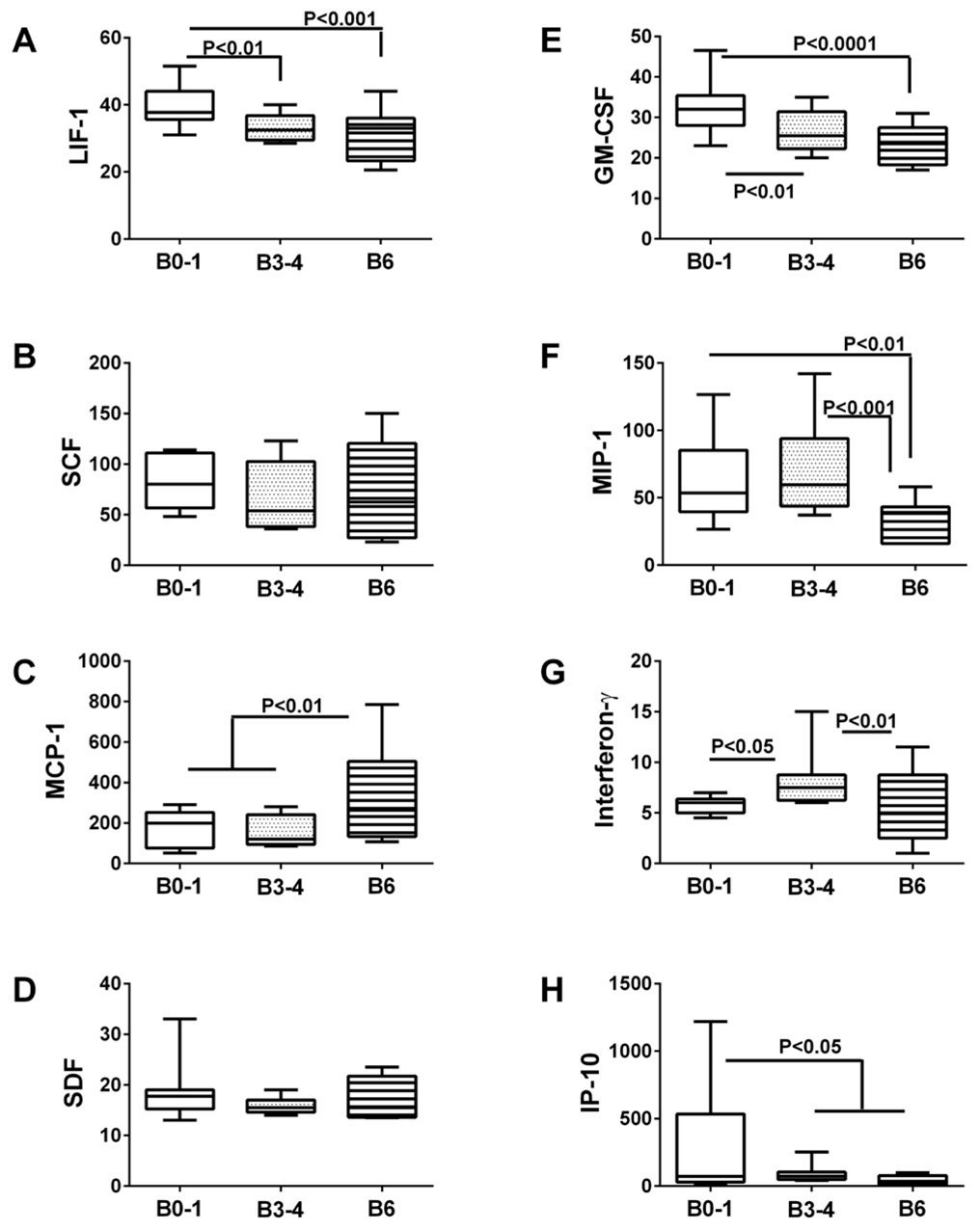
Inflammatory mediators in postmortem brains-2 Frontal lobe aqueous homogenates from subjects with Braak Stage (B) 0–1 (controls), B3–4 (moderate AD), or B6 (late AD) pathology (N=8/group) were used to measure (A) LIF-1, (B) scatter factor (SCF), (C) (MCP), (D) SDF, (E) GM-CSF, (F) MIP-1, (G) Interferon-γ, and (H) (IP-10) by multiplex bead-based ELISA. Immunoreactivity is expressed in fluorescence light units (FLU) normalized to protein content. Data were analyzed by one-way repeated measures ANOVA with post hoc Fisher significance tests.

Multiplex bead-based ELISAs were used to measure pro-inflammatory cytokines and chemokines in postmortem brain tissue (Figures 5 and 6), VF (Table 2), and CSF (Table 3). In brain, we observed four distinct trends pertaining to inflammatory mediators such that the levels of immunoreactivity: 1) declined progressively from Braak 0–1 to Braak 6 AD (LIF-1 (F=7.774; P=0.0013), GM-CSF (F=11.05; P=0.0001) and TRAIL (F=6.214, P=0.0041)); 2) were significantly but similarly reduced at Braak Stages 3–4 and 6 relative to control(IL-6 (F=6.17; P=0.0043) and IP10 (F=3.97, P=0.026)), 3) were similar for Braak 0–1 and 3–4, but significantly increased (MCP-1 (F=6.47, P=0.0034) and IL-16 (F=4.132, P=00225)), or decreased (IL-1β (F=6.85; P=0.0025), IL-18 (F=4.56; P=0.0158), and MIP-1(F=7.91. P=0.001)) at Braak Stage 6; and 4) higher at Braak Stage 3–4 compared with Braak Stage 0–1 and/or Braak Stage 6 (TNF-α(F=13.54, P<0.0001) and Interferon-γ (F=4.93, P=0.012)). In contrast, no significant differences were observed with respect to IL-8, IL-10, SCF, or SDF (Figures 5 and 6). Fisher post-hoc tests confirmed that inflammatory mediator levels were significantly suppressed in the late stages (IL-1β, IL-18, TRAIL, MIP-1) or both the intermediate and late stages (IL-6, LIF-1, GM-CSF, IP-10) of AD (Figures 5 and 6). However, selected inflammatory markers were increased either at Braak stage 3–4 (Interferon-γ and TNF-α), or Braak Stage 6 (IL-16 and MCP-1).

Analysis of postmortem VF samples demonstrated significantly reduced levels of IL-1β, IL-6, IL-10, TRAIL, MCP-1, and SDF in AD relative to control (Table 2). MIP-1, IP-10, GM-CSF, and LIF levels were also reduced in AD, but the differences did not reach statistical significance. Although other cytokines and chemokines were somewhat increased in AD (IL-8, IL-16, IL-18, TNF-α, and Interferon-γ), the differences did not reach statistical significance. Together, these findings suggest that AD is mainly associated with broad-based suppression of inflammatory cytokine responses, and modest increases in selected cytokines. The findings in postmortem VF were largely concordant with results obtained for brain tissue, with notable exceptions including IL-10, IL-18, MCP-1, and SDF, in which the results were contradictory.

In contrast to the findings in brain and VF, cytokine and chemokine levels were mainly increased in AD relative to control CSF (Table 3). Significant differences or trends corresponding to increased inflammation in AD were observed for IL-6, IL-16, LIF, and MCP-1. In addition, CSF levels of IL-8, SCF, MIP-1b, and IP-10 were also increased in AD, although the differences from control did not reach statistical significance. However, as observed in brain and VF, evidence for AD-associated CNS suppression of inflammatory mediators was marked by the significant reductions or trends in reduced levels of IL-1β, IL- 18, and TNF-α. The findings with respect to neuro-inflammation in CSF at the early and intermediate stages of AD were largely discordant with postmortem brain and VF results. On the other hand, the reduced levels of IL-1β and IL-18, and increased levels of IL-16 and MCP-1 in AD CSF did correspond with the postmortem findings in Braak 6 AD brains (Figures 5 and 6), and to some extent (IL-1β and IL-16) postmortem VF. Overall, the most informative and consistent correlate of AD was IL-1β suppression in brain, VF, and CSF. In contrast, neuro-inflammatory indices in CSF (early or moderate AD) seem not to inform about the levels of neuroinflammation in brains with Braak Stage 3–4 AD.

## Discussion

In AD, significant impairments in brain insulin signaling begin early in the clinical course and progress with disease severity [7,11,14]. A probable role for brain metabolic dysfunction in the pathogenesis of AD is further supported by the: 1) findings of cognitive impairment and neurodegeneration in experimental models of brain insulin/IGF resistance [6,49–53]; 2) halting or reversal of cognitive deficits by insulin, GLP-1 analog, or insulin sensitizer treatments in humans and experimental animals [18–27]; and 3) effectiveness of lifestyle changes for reducing insulin resistance and preserving cognition [54–57]. Despite these conceptual gains, AD diagnostic panels have not been revised to accommodate the metabolic/insulin resistance hypothesis, and instead remain largely focused on detecting altered levels of AβPP-Aβ and pTau in CSF. Data from neuroimaging and human brain studies strongly suggest that CNS metabolic indices, particularly those related to brain insulin signaling, could help with early detection of AD, monitoring the clinical course, and evaluating responses to treatment. This concept is reinforced by evidence of brain mitochondrial dysfunction which reflects significant perturbations in brain energy metabolism and begins early in the course of AD [43,44,58]. Finally, independent evidence suggests that consequences of, or co-factors in brain metabolic dysfunction, e.g. neuro-inflammation and oxidative stress, should be considered as they likely exacerbate or perpetuate the cascade of neurodegeneration [15,16,43]. The present study attempts to address these concepts by identifying clusters of additional potential biomarker indices that might be incorporated into AD diagnostic panels. The long-range objectives are to enhance sensitivity and specificity of AD detection, particularly in the early and most treatable phases of disease.

Consistent with the well-characterized neuropathology of AD and studies utilizing pTau and AβPP-Aβ CSF-based biomarker assays to diagnose or monitor AD [59–61], we detected increased levels of pTau and AβPP-Aβ in postmortem brains that had intermediate (Braak 3–4) or advanced (Braak 6) stages of AD. In addition, we observed increased levels of lipid peroxidation (4-HNE), advanced glycation end-products, and a trend for increased DNA damage (8-OHdG) with severity of AD. In sporadic AD, which was present in all AD cases in this study, Tau pathology is caused by aberrant activation of kinases that cause hyper-phosphorylation of the protein, leading to the formation of insoluble aggregates that undergo ubiquitination. Fibrillar aggregates of pTau promote oxidative injury and stress, which activate or exacerbate AβPP-Aβ pathology, neuro-inflammation, and cell death cascades [62]. Hyper-phosphorylated tau-associated lesions in AD are recognized as neurofibrillary tangles, dystrophic neurites, and neuropil threads, and their accumulations correlate with clinical severity of dementia. AβPP-Aβ pathology results from aberrant cleavage of AβPP, resulting in AβPP-Aβ fibril accumulation. Fibrillar aggregates of AβPP-Aβ undergo ubiquitination and promote oxidative stress, which can trigger or worsen Tau pathology, as well as promote cellular stress-related injury and inflammation [63]. In addition, soluble, diffusible and toxic AβPP-Aβ oligomers, which accumulate late in the course of AD, may have a role in AD progression due to neurotoxic injury [64] and inhibitory effects on insulin signaling [13].

Activation of glycogen synthase kinase 3β (GSK-3β) which has a pivotal role in promoting Tau hyper-phosphorylation [65], is a major consequence of impaired insulin signaling and insulin resistance [66–70]. Increased GSK-3β activation promotes oxidative stress and DNA damage [71], and oxidative stress is sufficient to increase AβPP-Aβ accumulation and Tau phosphorylation [72]. Likewise, insulin resistance promotes brain accumulations of pTau and AβPP-Aβ [49,52]; AβPP-Aβ toxic fibrils impair insulin signaling by down-regulating insulin receptors [13]. Together, these responses promote oxidative stress, neuro-inflammation, neurotoxicity, and synaptic dysfunction through a positive feedback loop that exacerbates insulin/IGF resistance [13,15]. Given this scenario, it is likely that multi-pronged biomarkers that detect different components of the neurodegeneration cascade will provide a more informative and sensitive diagnostic aid for detecting and monitoring AD at different stages of disease. Moreover, this strategy holds promise for early detection of AD, when the disease is most likely to respond to treatment. Lastly, simple, cost-effective biochemical and molecular tools are needed to objectively monitor therapeutic responses, as well as help to streamline postmortem diagnoses of AD.

Herein, we examined three clusters of potential biomarker for detecting AD neurodegeneration: insulin resistance, trophic factors, and inflammatory indices. The goals were to assess: 1) trends in AD-associated abnormalities in insulin resistance-related proteins; 2) AD-associated abnormalities in trophic factors that support different functions in the brain, including neuronal plasticity; and 3) patterns of neuro-inflammation in brain versus VF and CSF.

The results obtained with the multiplex gut hormone panel were reassuring with regard to the roles of insulin resistance and metabolic dysfunction in AD because they reported AD Braak stage declines in insulin and GLP-1, and increases in leptin. The significant reductions in GLP-1 correspond with the reduced insulin levels in brain and CSF. The trends with regard to GIP-1 and PYY were novel and suggest further studies should be done to characterize the full spectrum of AD-associated abnormalities in gut-pancreatic type polypeptides that occur over the course of disease. It is particularly noteworthy that the reductions in GLP-1 and GIP-1 could exacerbate the deficiencies in brain insulin levels and worsen impairments in brain insulin signaling, since both GLP-1 and GIP-1 are incretins with insulinotropic functions, and they are important regulators of glucose metabolism [73] that could be used therapeutically to treat cognitive impairment and neurodegeneration in AD [18,74]. Reduced levels of these polypeptides in AD correlate with decreased levels of insulin, thereby supporting their use in diagnostic panels as well as targeted therapy for AD. In addition, since many of the insulin resistance-related abnormalites in AD also occur in metabolic syndrome which contributes to cognitive decline [75], both CNS and systemic factors mediating brain metabolic dysfunction and insulin resistance could serve as therapeutic targets in AD [75–77].

Increased levels of leptin [78] and reduced levels of PYY [79] are features of obesity with peripheral insulin resistance [80]. The presence of similar abnormalities in AD brains suggests that leptin and PYY levels could also serve as indices of brain insulin resistance. Finally, ghrelin, a ligand for growth hormone secretagogue receptor, is downregulated in aging [81] and morbid obesity, which are insulin resistance states [82]. Therefore, reduced levels of ghrelin in AD correspond with brain aging and insulin resistance. The constellation of insulin, GLP-1, and GIP-1 deficiencies, together with alterations in other polypeptides that report brain insulin resistance is consistent with the hypothesis that AD represents Type 3 diabetes with combined features of insulin deficiency and resistance in the brain [12,14]. The consequences of these metabolic derangements were reflected by the increased levels of AGE, HNE, and 8-OHdG in postmortem AD brains.

The findings with respect to trophic factors were interesting because most of the trends showed increased expression levels in relation to AD severity. Exceptions were PDGF and VEGF, which declined, and GDNF, which was unchanged. Increased levels of NGF and BDNF in AD could reflect effects of receptor resistance, particularly given the impairments in neuronal plasticity and the roles these neurotrophins play in synaptic remodeling [83,84]. Despite its name, HGF is expressed in the brain, particularly the hippocampus and may have neurotrophic properties [85]. Its prominent localization in the CA3–CA4 regions of the hippocampus [85] where neurogenesis occurs [86], further suggests that HGF plays a key role in maintaining neuronal populations as well as mediating synaptic plasticity. The increased levels of HGF in AD brains corresponds with previous observations [87], and the somewhat higher levels in AD VF and CSF could also reflect HGF receptor resistance given the progressive impairments in neuronal plasticity that occur with AD progression. The findings with regard to β-FGF are entirely consistent with earlier observations in human postmortem brains [88]. Previous studies correlated increased β-FGF expression in AD with increased gliosis [88], which characteristically marks several aspects of neurodegeneration, including loss of neurons and fibers.

VEGF is expressed in microglia and endothelial cells. Alterations in VEGF expression occur in cerebral microvascular disease and in AD. In addition to its role in angiogenesis, VEGF has neuroprotective actions that may have relevance for treatment of AD and other neurodegenerative diseases [89]. In this regard, low VEGF levels have been shown to mediate neurodegeneration, which could be due to hypoperfusion or reduced neuronal protection from oxidative stress [89]. Therefore, reduced levels of VEGF in AD brains and VF could mark the presence and/or severity of neurodegeneration mediated by brain hypo-perfusion and neuronal death. This concept opens the door to additional treatment modalities for AD, as well as investigating whether the VEGF responses in AD are primary or secondary. For example, insulin and IGF-1 regulate expression of VEGF [90], and impairments in brain insulin and IGF-1 levels begin early in the course of AD [11].

Platelet-derived growth factor (PDGF) mediates β-γ secretase mediated cleavage of AβPP [91]. In addition, PDGF-BB, which is only expressed in neurons, is abundant in neurofibrillary tangles and associated with synaptic loss and dystrophic sprouting, whereas PDGF-AA is vascular associated [92] and mediates oligodendrocyte development. PDGF-AA, which was measured in the gut hormone panel, has an important role in myelin maintenance [93]. PDGF-A receptor is regulated by β-FGF [93], and PDGF regulates oligodendrocyte progenitor cells functions, including myelination [94,95]. Therefore, the reduced levels of PDGF in AD correspond with the previously demonstrated early loss of white matter and hypomyelination in this disease [96,97]. The fact that PDGF expression was reduced in AD brain and VF samples but not in the clinical CSF samples suggests that these abnormalities may be detectable in CSF only in the later stages of disease.

Neuro-inflammation remains a focus of research in AD because it occurs early in the course of disease [98], and already has been addressed in several clinical trials [99,100]. The failure to obtain conclusive evidence that anti-inflammatory measures are neuroprotective and can halt neurodegeneration most likely reflects the complexity and non-static nature of the problem. For example, inflammation may mediate disease at selected stages rather than throughout its clinical course. Multiplex ELISAs are an efficient way to simultaneously assess arrays of pro-inflammatory mediators in human subject material. A major unexpected finding was the broad-based suppression rather than activation of pro-inflammatory mediators in AD brains and postmortem ventricular fluid. In brain tissue, only IL-16, TNF-α, MCP-1, and Interferon-γ were elevated at either Braak Stage 3–4 or 6. For the other 12 cytokines/chemokines measured, 8 were expressed at significantly lower levels in brains with Braak Stage 6 or both Braak 3–4 and 6 AD relative to control. Similarly, in VF samples, only 5 of the 16 cytokines measured were elevated in AD but none of those differences were statistically significant. In contrast, in CSF, 4 cytokines were significantly elevated in AD, and 11 were moderately although not significantly elevated relative to control. However, since TNF-α and Interferon-γ expression were elevated in brains with Braak Stage 3–4 but not Braak 6 AD, and higher percentages of the inflammatory mediators were up-regulated in CSF as compared with brain or VF, conceivably the activation of neuro-inflammation occurs early in the course of AD, but as disease progresses, neuroinflammation subsides or is suppressed. The mechanisms and consequences of these responses are not known. However, the findings suggest that as a tool for evaluating AD diagnosis, severity, and responses to treatment, pro-inflammatory cytokines do not represent viable targets. On the other hand, the higher levels and profiles of inflammatory mediators in the clinical CSF samples from patients with probable AD suggest that anti-inflammatory therapeutic approaches may have value in the early stages of disease.

In conclusion, this study demonstrates the utility of evaluating indices of insulin resistance, neuronal plasticity, glial function, and oxidative stress in conjunction with pTau and AβPP-Aβ in CSF-based multiplex assays. This multi-pronged approach to assess different aspects of the neurodegeneration cascade will likely be more informative with respect to using streamlined biochemical and molecular assays for clinical as well as postmortem diagnoses, monitoring the clinical course of AD, and evaluating responses to treatment. This study suggests that the use of neuro-inflammatory markers will likely not be beneficial due to the transient nature of their activation in relation to disease severity. Future studies should assess the time course of shifts in biomarker indices in relation to cognitive decline and structural and functional neuroimaging abnormalities.

## Figures and Tables

**Table 1:** Trophic factors and cytokines probed in alzheimer brain, ventricular fluid and cerebrospinal fluid samples.

Trophic Factor	Abbreviation	Function
Basic Fibroblast Growth Factor	b–FGF	Present in basement membranes and sub-endothelial extracellular matrix. Regulates angiogenesis and cell survival, division, differentiation, and migration. Modulates nervous system development and wound healing.
Beta-Nerve Growth Factor	β–NGF	Neurotrophin family member that regulates survival and maintenance of sensory and sympathetic neurons. Implicated in neuronal growth, proliferation, differentiation and plasticity, as well as cognition. Functions through receptor tyrosine kinase.
Brain-Derived Neurotrophic Factor	BDNF	Neurotrophin family member that regulates synaptic transmission, activity-dependent plasticity and long-term potentiation in the hippocampus
Gastric Inhibitory Polypeptide	GIP-1	Glucose-dependent insulinotropic peptide and secretin family member. Stimulates insulin secretion from pancreatic beta-cells following food ingestion. GIP receptors expressed in hippocampus, olfactory bulbs, and cerebellum. Promotes neural progenitor cell proliferation.
Ghrelin	GHRL	Stimulates hunger, craving, and growth hormone secretion from the pituitary. Functions in opposite ways compared to leptin. Essential for cognitive adaptations in changing environments. Receptor expressed in hypothalamus.
Glial Cell Line-Derived Neurotrophic Factor	GDNF	Isolated from glioma cells. Member of TGF-β superfamily. Neurotrophic factor that exerts neuroprotective and differentiation effects on dopaminergic and motor neurons
Glucagon-like Peptide 1	GLP-1	Incretin whose secretion is regulated by nutrients, e.g. carbohydrate, protein, and lipid. Promotes glucagon-dependent stimulation of insulin secretion, and survival and proliferation of pancreatic beta cells. Enhances insulin sensitivity and satiety.
Hepatocyte Growth Factor	HGF	Typically secreted by mesenchymal cells with actions on epithelial and endothelial cells. Mediates embryogenesis. Stimulates mitogenesis, cell motility, matrix invasion and angiogenesis via c-MET receptor tyrosine kinase. Regulates VEGF. Neuroprotective for cortical and hippocampal neurons during aging and ischemic injury.
Insulin	INS	Reduces blood glucose. Increases cellular permeability to monosaccharides, amino acids and fatty acids. Increases rates of glycolysis, pentose phosphate cycle, and glycogen synthesis in liver.
Leptin	LEP	Produced in adipocytes and regulates brain energy intake and expenditure, metabolism, and behavior.
Pancreatic Polypeptide (Human)	PP	Polypeptide secreted by PP endocrine cells in pancreas in response to hypoglycemia, fasting, or protein meal and decreased by glucose infusion or somatostatin. Closely related to neuropeptide Y and PP.
Peptide YY (tyrosine-tyrosine)	PYY	Secreted by intestinal L cells in response to feeding. Reduces appetite. Also produced in brainstem neurons, pancreatic islets. Improves nutrient absorption by slowing gastric motility and emptying.
Platelet-derived Growth Factor-AA	PDGF-AA	Regulates cell growth and angiogenesis, and mitogenic for glial and mesenchymal cells. Signals through PI3 Kinase to regulate cell growth and motility, tissue remodeling, differentiation, and migration. Maintains proliferation of oligodendrocyte progenitor cells.
Vascular Endothelial Growth Factor	VEGF	Stimulates angiogenesis and vasculogenesis and endothelial cell growth. Inhibits apoptosis and induces vascular permeability, revascularization of injured tissue, endothelial cell migration and proliferation.

Cytokine/Chemokine	Abbreviation	Function
Granulocyte Macrophage Colony-Stimulating Factor	GM-CSF	Stimulates the growth and differentiation of hematopoietic precursor cells from various lineages, including granulocytes, macrophages, eosinophils and erythrocytes.
Interferon-gamma	IFN-γ	Produced by innate NK cells, acquired antigen-specific cytotoxic CD4+ and effector CD8+ T cells. Activates macrophages and critical for innate and adaptive immune responses to intracellular pathogens, tumor control, and inhibition of viral replication.
Interferon-Gamma-Induced Protein	IP-10	Produced by various cell types including monocytes, endothelial cells, fibroblasts, and keratinocytes. Induced by IFN-γ and TNF-α. Chemoattractant for activated T cells.
Interleukin-1, Beta	IL-1β	Produced by activated macrophages; mediates inflammatory responses, cell proliferation, and apoptosis. Induces Cox-2 in CNS, causing inflammatory pain
Interleukin-6	IL-6	Secreted by T cells and macrophages; triggers inflammation, acute phase response, fever. Anti-inflammatory effects include inhibiting TNF-α and IL-1, and activating IL-1Rα and IL-10.
Interleukin-8	IL-8	Made by macrophages and some epithelial and endothelial cells; Role in innate immune response. Major role in chemotaxis of neutrophils. Also mediates inflammatory response and angiogenesis.
Interleukin-10	IL-10	Produced by monocytes. Pleiotropic cytokine. As an anti-inflammatory cytokine, it inhibits macrophage and dendritic cell function, suppresses TNF-α. Acquires pro-inflammatory activity during immune response with IFN-α stimulation
Interleukin-16	IL-16	Secreted by lymphocytes. Pleiotropic cytokine. Functions as a chemo-attractant (CD4+ cells), modulates T cell activation, and inhibits HIV replication.
Interleukin-18	IL-18	Produced by macrophages and monocytes. Pro-inflammatory cytokine interacts with IL-12 to induce cell-mediated immune response with microbial infection and LPS, inducing severe inflammatory reactions. Stimulates NK and T cell release of IFN-γ, which activates macrophages. Inhibits IL4-dependent IgE, enhances B cell production.
Leukemia Inhibitory Factor	LIF	Induces myeloid cell differentiation, neuronal cell differentiation, and acute-phase protein synthesis.
Macrophage Inflammatory Protein 1 Beta	MIP-1β	Produced by macrophages. CCL4 chemokine that generates local inflammatory responses induces superoxide production by neutrophils. Chemotactic activity for lymphocytes, macrophages, NK cells, and monocytes with inflammation; down-regulates CCR5, inhibiting HIV-1 blocking.
Monocyte Chemotactic Protein-1	MCP-1	Expressed in monocytes, vascular endothelial cells, smooth muscle cells. CCL2 chemokine induces monocyte attraction, and degranulation of basophils with histamine release. Induced by IL-1, TNF-α, PDGF, TGF-β, and LIF
Stem Cell Factor	SCF	Regulates cell survival and proliferation, hematopoiesis, stem cell maintenance, gametogenesis, and mast cell development, migration and function. Promotes phosphorylation of PIK3R1 and activation of AKT1. Aids in activation of RAS, RAF1 and the MAP kinases MAPK1/ERK2 and/or MAPK3/ERK1
Stromal Cell-Derived Factor-1α	SDF-1α	Expressed ubiquitously, except in blood cells. Small cytokine member of CXCL12 family of chemokines. Activates leukocytes due to strong chemotactic effects. Induced by pro-inflammatory stimuli, e.g. TNF-α and IL-1β.
TNF-Related Apoptosis Inducing Ligand	TRAIL	Secreted by macrophages, monocytes, neutrophils, T cells, NK cells after stimulation with LPS. CD4+ cells secrete TNF-α. Also made by astrocytes, microglial cells, smooth muscle cells, and fibroblasts. Mediates systemic inflammation, inhibits viral replication, and inhibits tumorigenesis.
Tumor Necrosis Factor-Alpha	TNF-α	Expressed broadly in tissues. Cytokine induces proapoptotic caspase activity by up-regulating pro-apoptotic Bcl proteins. Causes apoptosis in hepatocytes, neural cells, and thymocytes

**Table 2:** Trophic Factor and Cytokine Levels in Post-mortem Ventricular Fluid.

Protein	Control	Alzheimer	P- Value
**Trophic Factors**			
β-NGF	41.76 ± 4.26	48.60 ± 8.14	
PDGF-AA	111.52 ± 9.94	59.73 ± 4.69	0.0002
HGF	1305.97 ± 299.12	3887.81 ± 691.31	0.0001
VEGF	195.74 ± 12.52	120.72 ± 9.74	0.0003
β-FGF	948.78 ± 81.77	546.07 ± 46.79	0.0005
**Cytokines**			
IL-1β	123.40 ± 44.97	34.23 ± 7.75	0.028
IL-6	316.64 ± 108.93	126.60 ± 91.79	0.009
IL-8	806.30 ± 321.41	929.34 ± 185.10	
IL-10	18.20 ± 1.19	13.95 ± 0.97	0.008
Il-16	2769.95 ± 244.39	3058.46 ± 219.60	
IL-18	365.60 ± 48.50	672.60 ± 218.45	
TNF-α	12.95 ± 1.03	15.93 ± 2.72	
TRAIL	61.14 ± 13.65	25.67 ± 2.84	0.01
LIF-1	58.19 ± 3.02	51.27 ± 3.06	
SCF	30.14 ± 3.14	28.14 ± 1.29	
MCP-1 (CCL2)	1793.48 ± 559.87	1123.61 ± 496.75	0.001
SDF (CXCL12)	79.25 ± 11.76	29.75 ± 2.76	<0.0001
GM-CSF	39.05 ± 1.29	33.54 ± 2.83	
MIP-1	2279.33 ± 791.99	1177.78 ± 142.71	
IFN-γ	6.66 ± 0.37	6.93 ± 0.55	
IP-10	734.19 ± 479.61	398.13 ± 96.21	

Postmortem ventricular fluid samples from aged controls or patients with documented late-stage AD (N=10/group) were used to measure immunoreactivity to trophic factors by direct binding ELISAs, and cytokines by multiplex bead-based ELISAs. Immunoreactivity was normalized to protein concentration and data are expressed as mean ± SEM of fluorescence light units (arbitrary). Inter-group comparisons were made with the Student T-test. Significant P values are listed in the right column.

**Table 3:** CSF Trophic Factor and Cytokine Levels in Probable AD.

Protein	Control	Alzheimer	P- Value
**Trophic Factors**			
β-NGF	5.69 ± 0.77	4.68 ± 0.17	P=0.10
PDGF	19.88 ±1.27	19.19 ± 0.36	
HGF	98.00 ± 7.09	111.5 ± 10.44	
VEGF	89.00 ± 2.15	103.9 ± 2.45	P<0.0001
β-FGF	89.81 ± 2.50	81.81 ± 2.29	P=0.025
**Cytokines**			
IL-1β	6.25 ± 0.62	4.69 ± 0.17	P=0.018
IL-6	21.00 ± 3.05	31.19 ± 4.42	P=0.067
IL-8	68.25 ± 8.50	77.00 ± 7.70	
IL-10	10.81 ± 0.51	11.50 ± 0.43	
Il-16	14.75 ± 0.71	17.44 ± 0.59	P=0.0035
IL-18	11.81 ± 1.98	8.94 ± 0.83	P=0.095
TNF-α	7.31 ± 0.66	6.38 ± 0.26	0.098
TRAIL	14.94 ± 1.05	14.13 ± 0.39	
LIF	36.56 ± 1.82	41.56 ± 1.34	P=0.017
SCF	57.63 ± 6.13	64.0 ± 5.08	
MCP-1 (CCL2)	2971 ± 80.11	3165 ± 118.3	P=0.093
SDF (CXCL12)	21.88 ± 2.11	22.06 ± 1.51	
GM-CSF	28.38 ± 1.27	29.00 ± 0.71	
MIP-1β	85.94 ± 10.94	98.13 ± 6.90	
IFN-γ	3.75 ± 0.62	3.00 ± 0.05	
IP-10	1578 ± 155.9	1739 ± 121.0	

CSF samples from controls (N=12) or patients with clinically diagnosed probable AD (N=16; confirmed by postmortem exam) were used to measure immunoreactivity to trophic factors by direct binding ELISAs, and cytokines by multiplex bead-based ELISAs. Immunoreactivity was normalized to protein concentration and data are expressed as mean ± SEM of fluorescence light units (arbitrary). Inter-group comparisons were made with the Student T-test. Significant P values are listed in the right column.
